# CORRELATES OF PHYSICAL ACTIVITY HISTORY IN PARTICIPANTS OF THE BALTIMORE LONGITUDINAL STUDY OF AGING

**DOI:** 10.1093/geroni/igac059.1232

**Published:** 2022-12-20

**Authors:** Ann Moore, Eleanor Simonsick, Bennett Landman, Luigi Ferrucci

**Affiliations:** TGB NIA NIH, Baltimore, Maryland, United States; National Institute on Aging, Baltimore, Maryland, United States; Vanderbilt University, Nashville, Tennessee, United States; National Institute on Aging, Baltimore, Maryland, United States

## Abstract

Physical activity across the life course contributes to physical function and health in later life. Here we characterize a measure of physical activity history, newly implemented in participants of the Baltimore Longitudinal Study of Aging (n = 690). Participants selected one of four levels to describe activity during each decade of life from age ten to the present. Recalled levels of physical activity are positively associated with activity assessed in current and prior decade study visits, suggesting that the recalled estimates are consistent with historic activity. A summary measure based on ranking activity patterns was associated with measures of physical performance, muscle and fat areas quantified from computed tomography images as well as some indicators of homeostatic dysregulation (p <.05). The observed associations suggest that an estimate of physical activity across decades provides complementary information to estimates of current activity and reemphasizes the importance of consistently engaging in physical activity.

